# Lobectomy with ECMO Support in an Infant Who Developed Pulmonary Interstitial Emphysema Following Repair of Hypoplastic Aortic Arch

**DOI:** 10.21470/1678-9741-2018-0135

**Published:** 2018

**Authors:** Michael Magarakis, Dao M. Nguyen, Alejandro E. Macias, Eliot R. Rosenkranz

**Affiliations:** 1 Department of Surgery; Division of Cardiothoracic Surgery; Cardiac Surgery section, Jackson Memorial Hospital - University of Miami Miller School of Medicine, Miami, USA.; 2 Department of Surgery, University of Medicine and Health Sciences, Miami, USA.; 3 Department of Surgery; Division of Cardiothoracic Surgery; Pediatric and Congenital Cardiac Surgery Section Jackson Memorial Hospital - University of Miami Miller School of Medicine, Miami, USA.

**Keywords:** Extracorporeal Membrane Oxygenation, Infant, Newborn, Diseases, Pulmonary Emphysema, Respiratory Distress Syndrome, Newborn, Aorta, Thoracic/Abnormalities/Surgery

## Abstract

Pulmonary interstitial emphysema (PIE) is a common problem in premature neonates with respiratory distress syndrome. This condition is often related to barotrauma caused by mechanical ventilation or continuous positive airway pressure applied to low birth weight neonates. The clinical diagnosis can be challenging. However, after proper diagnosis, several interventions are available for successful management. We describe an infant who developed severe PIE with recurrent pneumothoraces and development of a persistent bronchopleural fistula shortly after repair of a hypoplastic aortic arch and description of successful lobectomy with the assistance of extracorporeal support (ECMO).

**Table:** 

Abbreviations, acronyms & symbols	
BPF	= Bronchopleural fistula
ECMO	= Extracorporeal membrane oxygenation
HFOV	= High frequency oscillatory ventilation
PIE	= Pulmonary interstitial emphysema
POD	= Postoperative day
SVT	= Supraventricular tachycardia

## CASE REPORT

The patient was born at 38 weeks of gestation via C-section delivery due to prolonged labor. Prenatal history was notable for a fetal echocardiogram, which demonstrated a moderately dilated and hypertrophied right ventricle and a hypoplastic aortic arch. The fetus had episodes of narrow complex tachycardia with heart rate ranging from 180-305 bpm. There was no evidence of hydrops. Postnatally, an electrocardiogram demonstrated periods of supraventricular tachycardia (SVT); and, an echocardiogram confirmed the presence of a hypoplastic aortic arch, mildly hypoplastic left ventricle, and secundum atrial septal defect. Prostaglandin infusion was started to maintain ductal patency. The next day, the patient developed ectopic atrial rhythm, which was followed by recurrent episodes of SVT with a heart rate in the 300s; the last episode required acute administration of adenosine and prevention of subsequent episodes was achieved with propranolol. On the fourth day of life, he underwent repair of his hypoplastic aortic arch by homograft patch enlargement and primary closure of his ASD – total cardiopulmonary bypass time was 153 minutes, and cross-clamp time was 90 minutes. After separation from bypass, the lung compliance was relatively poor - necessitating high inspiratory pressures. The hyper-inflated lungs led to leaving the chest open, followed by delayed closure on the third postoperative day (POD). A few hours postoperatively, the patient again had episodes of SVT: at this time, suspicion arose of a concealed Wolff-Parkinson-White pathway, which was managed with an amiodarone drip with eventual addition of flecainide.

**Fig. 1:**
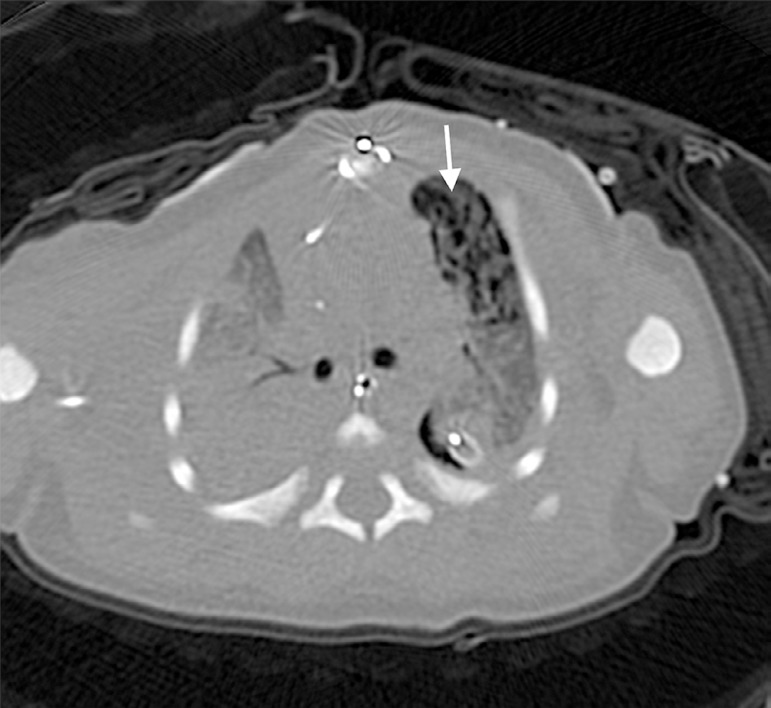
Transverse computed tomography displaying left upper lobe pulmonary emphysema (white arrow).

Postoperatively, the lung compliance improved: on POD 8, mediastinal and pleural chest tubes were removed. Immediately after, the patient developed left-sided tension pneumothorax, which was treated by insertion of a pigtail catheter. A second chest tube was required the following day due to a recurrent left pneumothorax with persistent air leak. The patient was placed on high frequency oscillatory ventilation (HFOV) to reduce peak and mean airway pressures. A computed tomography of the chest was consistent with pulmonary interstitial emphysema (PIE) of the left upper lobe with an associated bronchopleural fistula (BPF) (Figure 1). Due to repeated pneumothoraces with conservative management, we proceeded with left upper lobectomy to control the fistula. Preoperatively, the patient was placed in the right lateral decubitus position, simulating the planned surgical procedure, which resulted in inadequate ventilation despite adjustment of mechanical support.

**Fig. 2:**
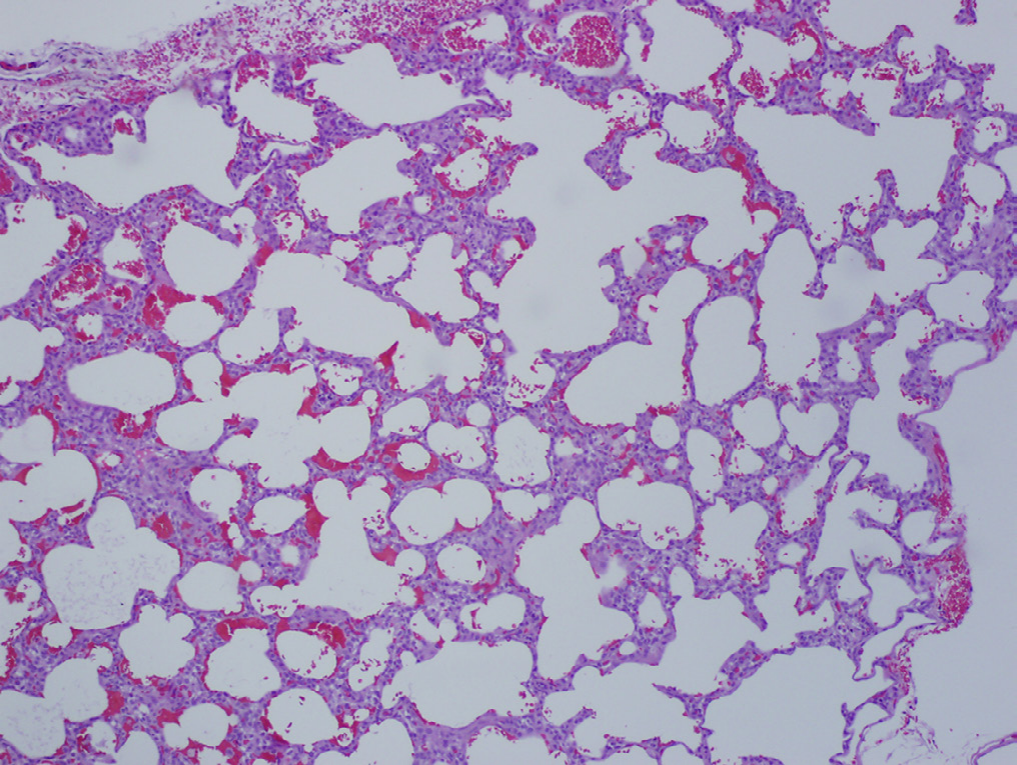
Hematoxylin and eosin-stained left upper lobe lung sections showing dilated air spaces with interstitial fibrosis and vascular congestion (x20 magnification).

Due to the patient's hemodynamic lability, we attempted to insert a veno-venous cannula into the jugular vein; however, the vessel was too small to accept the smallest cannula. As such, a single lumen venous line was inserted and secured and carotid cannula was inserted for full support. A left posterolateral thoracotomy was made, and the fourth intercostal space was entered. After inspection of the lung, a clear demarcation where the upper lobe had multiple cysts and lingula, as well as evidence of emphysema were noted. The upper lobe pulmonary vein was carefully exposed as was the upper lobe pulmonary artery; however, we felt that an anatomic resection would be too prolonged given the patient's tenuous condition. As such, two staple lines were applied across the division point between the upper lobe and the lingula taking care to avoid injury to the superior vein or the pulmonary artery. A second application of staples allowed removal of the involved upper lobe. The left upper lobectomy and pleural tent successfully controlled the BPF. Histologic inspection of the lung demonstrated numerous cysts localized to the left upper lobe with sparing of the remaining left lung parenchyma (Figure 2). A BPF was demonstrated originating from the anteromedial segment of the upper lobe.

Post-operatively, he was maintained on HFOV and was weaned and separated from extracorporeal membrane oxygenation (ECMO) on post-lobectomy day 6. On post-lobectomy day 21, patient transitioned to conventional ventilator and was extubated. He has done well 8 months postoperatively without respiratory symptoms, need for supplemental oxygen, or tachyarrhythmias.

## DISCUSSION 

Prematurity and low birth weight (<1500 grams) are known risk factors for developing PIE; the perivascular connective tissue in the lung is more abundant in preterm infants, which allows for air trapping in the perivascular space^[1]^.

Infants who suffer from PIE are at risk for developing other pulmonary complications such as BPF with persistent air leak and compromised ventilation due to compression of healthy lung parenchyma by large bullae^[2]^. Different treatment approaches have been developed: conservative and surgical interventions. Conservative measures consist of ventilation, positioning, selective intubation, and steroids - HFOV can be used successfully, as was used in our case^[3-6]^.

In situations where conservative management fails, surgery can be curative if the disease involves a segment of lung that can be removed without major pulmonary complications for the newborn. In our case, PIE involved only the left upper lobe, and left upper lobectomy was curative.

In our case, the patient was not premature and had a normal preoperative pulmonary status with no respiratory compromise. He developed severe PIE of his left upper lobe shortly after his aortic arch repair. When we noticed lung compliance was suboptimal at the end of the case, we suspected high mean airway pressures caused PIE. The chest was left open in an effort to improve chest mechanics and allow for better lung expansion. The use of ECMO was then used as a bridge for a safe left upper lobectomy.

## CONCLUSION

Performing a lobectomy with the successful assistance of ECMO is infrequent. Lung resection, with or without extracorporeal support, should be a viable alternative in patients with persistent isolated PIE, especially those that have suffered complications such as pneumothorax, or persistent air leak.

**Table:** 

Authors' roles & responsibilities	
MM	Substantial contributions to the conception or design of the work; or the acquisition, analysis, or interpretation of data for the work; drafting the work or revising it critically for important intellectual content; final approval of the version to be published
DMN	Agreement to be accountable for all aspects of the work in ensuring that questions related to the accuracy or integrity of any part of the work are appropriately investigated and resolved; final approval of the version to be published
AEM	Substantial contributions to the conception or design of the work; or the acquisition, analysis, or interpretation of data for the work; drafting the work or revising it critically for important intellectual content; final approval of the version to be published
ERR	Substantial contributions to the conception or design of the work; or the acquisition, analysis, or interpretation of data for the work; drafting the work or revising it critically for important intellectual content; final approval of the version to be published
